# Clinicoradiological Profile and Functional Outcome of Acute Cerebral Venous Thrombosis: A Hospital-Based Cohort Study

**DOI:** 10.7759/cureus.17898

**Published:** 2021-09-12

**Authors:** Matteo Foschi, Lucia Pavolucci, Francesca Rondelli, Giulia Amore, Luca Spinardi, Rita Rinaldi, Elisabetta Favaretto, Luca Favero, Michele Russo, Umberto Pensato, Matteo Benini, Valentina Barone, Maria Guarino

**Affiliations:** 1 Department of Neuroscience, Neurology Unit, S.Maria delle Croci Hospital of Ravenna – AUSL Romagna, Ravenna, ITA; 2 Department of Medical and Surgical Sciences, University of Bologna, Bologna, ITA; 3 Neurology Unit, S.Orsola-Malpighi University Hospital of Bologna, Istituto di Ricovero e Cura a Carattere Scientifico (IRCCS) Istituto delle Scienze Neurologiche di Bologna, Bologna, ITA; 4 Department of Biomedical and Neuromotor Sciences, University of Bologna, Bologna, ITA; 5 Department of Experimental Diagnostic and Specialty Medicine, Neuroradiology Unit, S.Orsola-Malpighi University Hospital of Bologna, Bologna, ITA; 6 Neurology Unit, S.Orola-Malpighi University Hospital of Bologna, Istituto di Ricovero e Cura a Carattere Scientifico (IRCCS) Istituto delle Scienze Neurologiche di Bologna, Bologna, ITA; 7 Department of Angiology and Blood Coagulation, Angiology and Blood Coagulation Unit, S.Orsola-Malpighi University Hospital of Bologna, Bologna, ITA; 8 Department of Health Management, S.Orsola-Malpighi University Hospital of Bologna, Bologna, ITA; 9 Department of Cardiovascular Diseases, Division of Cardiology, S.Maria delle Croci Hospital of Ravenna, AUSL Romagna, Ravenna, ITA; 10 Department of Biomedical and Neuromotor Sciences, Univesity of Bologna, Bologna, ITA

**Keywords:** outcome, prognosis, neuroimaging, headache, cerebral venous thrombosis

## Abstract

Introduction

Acute cerebral venous thrombosis (CVT) may result in a variety of clinical presentations, with headache being the most common. The relationship between clinical and neuroradiological characteristics in acute CVT patients is still not univocally characterized.

Materials and methods

We enrolled 32 consecutive acute CVT patients admitted to our emergency department from January 1, 2012, to June 30, 2019. Clinicoradiological associations and their relationship with the functional outcome at the discharge were tested.

Results

Headache was the presenting symptom in 85% of patients, more frequently subacute (82%), new-onset (67%), with unusual features in respect to prior headache episodes (100%), and associated with concomitant neurological symptoms/signs (74%). Patients with holocranial headache showed more frequent venous ischemia (VI) compared to those with bilateral and unilateral headache (50% vs. 20% vs. 0%, respectively; p=0.027). Patients with concomitant neurological defects had a higher prevalence of VI (50.0% vs. 15.0%; p=0.049) and superior sagittal sinus thrombosis (67% vs. 30%; p=0.043) than those without. Vomit was more frequently observed in patients with straight sinus thrombosis (67% vs. 8%; p=0.005). Increasing age and VI were independently associated with poor (modified Rankin scale (mRS) 2-5) functional outcome (odds ratio (OR) = 1.081, 95% confidence interval (CI) 1.004-1.165; p=0.038 and OR = 12.089, 95% CI 1.141-128.104; p=0.039, respectively).

Conclusions

Our study confirms and enriches available data on the clinicoradiological profile of patients with acute CVT and suggests that increasing age and venous ischemia are independently associated with poor outcomes.

## Introduction

Cerebral venous thrombosis (CVT) is a rare cerebrovascular disease with an estimated incidence of 5/1,000,000 people per year [1]. Although accounting for less than 1% of all strokes [1-2], CVT-related complications (e.g. venous ischemia (VI), intracranial hypertension (IH), parenchymal hemorrhage (PH), subarachnoid hemorrhage (SAH)) account for important morbidity and mortality rates (about 15%) [3]. CVT has a three-fold higher incidence in women younger than 50 years old, reflecting sex-specific risk factors such as pregnancy, contraceptive use, and estrogen fluctuations [1-2,4-5]. Other less common risk factors include genetic or paraneoplastic thrombophilia, infectious or autoimmune disorders, and traumatic skull injury [1-2,4-5]. CVT can present with a multitude of neurological symptoms depending on many factors such as age and the anatomical location of the thrombus [6-7]. Headache is notoriously the most common symptom of CVT (up to 90% of all cases) [7] and can result from either mechanical stretching of trigeminal nerve fibers in the walls of the occluded sinus or from cortical and dural inflammation [1-2]. However, CVT-related headache location and characteristics are extremely heterogeneous, ranging from thunderclap pain to less specific features (e.g. throbbing pain with nausea, vomit, phono/photophobia-mimicking migraine, or other primary headache disorders [6,8]. Few studies attempting to identify a recurrent headache pattern have failed to provide univocal data [6-7] so that the clinical suspicion of acute CVT still relies on the concomitant presence of risk factors and neuroimaging findings [1-2,4-8]. Furthermore, the relationship between headache and neuroimaging findings has been not univocally characterized, and the only reported association is between occipital pain and sigmoid sinus thrombosis [9-10].

With this background, our study aimed to investigate the clinicoradiological profile and to identify potential associations with the functional outcome in a retrospective cohort of CVT patients.

## Materials and methods

Study design and participants

We retrospectively enrolled all consecutive patients aged ≥ 18 years old admitted to the emergency department (ED) of S.Orsola-Malpighi University Hospital of Bologna and discharged between January 1, 2012, and June 30, 2019, with a diagnosis of CVT (study period = 90 months). Additionally, we interrogated our hospital database in order to check the inclusion of all CVT cases with the following International Classification of Diseases, Ninth Revision Clinical Modification (ICD-9 CM) codes: 437.6 (non-pyogenic thrombosis of intracranial venous sinus, 325 (phlebitis and thrombophlebitis of intracranial venous sinuses), and 671.5 (other phlebitis and thrombosis in pregnancy and puerperium). Overall, patients underwent several neurological evaluations during admission. However, medical records were also extracted from discharge reports and checked by an expert vascular neurologist (MG) to confirm the diagnosis. Clinical, neuroimaging, treatment, and outcome data were extrapolated from medical records, neurological, angiological visits, and the mortality registry. A blinded expert neuroradiologist (LS) revaluated and classified neuroimaging findings. All patients underwent at least ≥1 clinical follow-up visit after discharge.

Definitions, classifications, and diagnostic procedures

Headache was classified according to the time from the onset as acute (<48 hours), including thunderclap headache (sudden onset with maximum intensity within 1 min), subacute (48 hours - 1 week), and chronic (>1 week). Pain intensity was assessed using the numeric rating scale (NRS) and divided into mild (NRS ≤4), moderate (NRS 5-7), and severe (NRS >8). Pain was defined as refractory to medications when persisting with moderate-to-severe intensity (NRS 5-10) despite the administration of adequate analgesic treatment. Unenhanced brain CT scan was considered consistent with CVT in the presence of a hyper-attenuating clot in the cerebral vein system, signs of VI (infarct not conforming to a conventional artery territory or spanning ≥1 territory with sparing of the cortex, multiple or bilateral lesions with/without hemorrhage) [11]. CVT diagnosis was confirmed according to the American Health Association/American Stroke Association (AHA/ASA) international guidelines for CVT diagnosis and management [5]. Recanalization following CVT was classified as complete, partial, or absent according to the degree of residual flow at the follow-up CT/MR venography. Functional outcome was measured according to the modified Rankin scale (mRS) score and classified as good (mRS 0-1) or poor outcome (mRS 2-5).

Statistical analysis

Patients were stratified into different groups based on clinical variables. Demographics and neuroimaging patterns were compared between patients dichotomized according to the presence or absence of the specific clinical feature in each of these groups. Categorical variables were summarized as frequencies and percentages while continuous variables were presented as median (interquartile range - IQR). Categorical variables were compared using the chi-square test or Fisher exact test, as appropriate. The student t-test for unpaired samples or the Mann-Whitney U test was used to compared normally or non-normally distributed variables, respectively. Univariate logistic regression analysis was performed to study the association of demographics, clinical features, and neuroimaging findings with poor functional status. Subsequently, variables showing a statistically significant association with the outcome at univariate analysis were included in a multivariate logistic regression model to establish independent associations with a poor functional outcome at discharge. Results were presented as odds ratio (OR) with a 95% confidence interval (CI). A p-value of ≤0.05 was considered statistically significant. Statistical analysis was performed with SPSS software, version 21.0 (IBM Corp., Armonk, NY).

Data availability

Pseudonymized participant data and results of analyses not included in the article will be made available upon request to the corresponding author.

## Results

Thirty-four patients aged >18 years old with a diagnosis of acute CVT were included in the study. Two patients were excluded because of secondary referral to our hospital for CVT complications. Therefore, we included 32 patients in the study. Since our hospital ED services covered about 250,000 persons, the estimated CVT incidence resulted to be about four patients/250,000 inhabitants per year (16/1,000,000 per year). The median age of our population was 41 years (IQR 26-49) with a clear female predominance (75%). All patients underwent cell blood count, D-dimer, fibrinogen blood levels, and screening for known causes of hereditary thrombophilia (hyperhomocysteinemia, antiphospholipid antibodies, antithrombin III deficiency, protein S/C deficiency, factor V Leiden mutation, prothrombin mutation, activated protein C resistance). Among gender-specific risk factors, the assumption of oral contraceptives was the most prevalent condition (67%), followed by pregnancy (4%). The presence of hereditary thrombophilia was the most frequent non-gender-specific risk factor (9%). For five patients (16%), we could not identify any possible prothrombotic factor. Demographic characteristics and frequency of risk factors are reported in Table 1.

**Table 1:** Demographic characteristics and frequency of risk factors IQR = interquartile range § Malignancies included a patient with pancreatic adenocarcinoma and a patient with a history of essential thrombocythemia (positive genetic testing for JAK V617F mutation)

Demographic characteristics	
Age – Median (IQR)	41 (26 – 49)
Sex: Female – N (%)	24/32 (75)
Risk factors	N (%)
Gender-specific (female)	17/24 (71)
Oral contraceptives	16/24 (67)
Pregnancy	1/24 (4)
Non-gender-specific	21/32 (66)
Hereditary thrombophilia	3/32 (9)
Factor V Leiden (heterozygous mutation)	2/32 (6)
Prothrombin G20210A (homozygous mutation)	1/32 (3)
Maxillofacial infections	4/32 (13)
Mastoiditis	2/32 (6)
Dental abscess	1/32 (3)
Odontogenic meningitis	1/32 (3)
Malignancy	2/32^§^ (6)
Lupus anticoagulants (LAC)	2/32 (6)
Hyperhomocysteinemia	2/32 (6)
Hyperfibrinogenemia	2/32 (6)
Thrombocythemia	2/32 (6)
Recent lumbar puncture (<1 month)	1/32 (3)
Traumatic skull injury	1/32 (3)

Clinical presentation

The median time between symptoms onset and neurological evaluation in the ED was four days (IQR 3-11). Neurological signs at presentation favored early ED admission (median time 2 days, IQR 2-3). Baseline mRS was 0 for all but a single patient with mRS 1. Headache was present in most of the patients at admission (27/32, 85%), with a de novo presentation in 67% of cases, and in all (100%) cases, it was the presenting symptom of acute CVT. Among patients presenting with a headache, 33% had a prior headache history, but they all experienced unusual pain as the inaugural CVT symptom. Patients presenting with a headache tended to be younger in comparison with subjects without a headache (median 39, IQR 25-46 vs. 61, IQR 53-64 respectively; p=0.087). In most cases, the headache presented with concomitant neurological symptoms (74%) while the remaining seven patients had isolated headaches (26%). No patient reported a thunderclap headache, with the majority (81%) showing a subacute onset. The median NRS was 6 (IQR 4-8). Besides headache, two cases presented with cavernous sinus syndrome (6%) and two cases with a decreased level of consciousness (6%). Only one patient (3%) presented with isolated focal seizures. Clinical features are displayed in Table 2.

**Table 2:** Clinical characteristics IH = intracranial hypertension *: different features in respect to prior headache episodes

	N (%)
Clinical presentation with headache	27/32 (85)
Unusual headache*	9/27 (33)
New-onset headache	18/27 (67)
Chronological presentation	
Thunderclap	0/27 (0)
Acute (<48 hours)	4/27 (15)
Subacute (48 hours - 1 week)	22/27 (81)
Chronic (>1 week)	1/27 (4)
Headache characteristics	
Mild	8/27 (30)
Moderate	10/27 (37)
Severe	9/27 (33)
Persistent and refractory to common analgesic	22/27 (81)
Isolated headache	7/27 (26)
Headache associated with other neurological signs/symptoms (“headache plus”)	20/27 (74)
+ Focal neurological defects	12/20 (60)
+ Focal and/or generalized seizures	1/20 (5)
+ ≥ 1 sign/symptom of IH (visual loss and/or 6th cranial nerve palsy and/or tinnitus)	7/20 (35)
Headache location	
Holocranial	12/27 (57)
Unilateral	10/27 (37)
Ipsilateral to the involved sinus	9/10 (90)
Bilateral (“band-like”)	5/27 (19)

Neuroimaging findings

All patients underwent an urgent unenhanced head CT scan, which resulted positive for signs suggestive of CVT in 83% of cases. In all cases (100%) the diagnosis of CVT was confirmed by either brain CT (59%) or MR venography (41%). Nine out of thirty-two patients (28%) showed CT signs of VI, while hemorrhagic complications were observed in six patients (19%). The median (IQR) number of involved sinuses was 2 (2-3). Neuroimaging findings are presented in Table 3.

**Table 3:** Neuroimaging findings CVT = cerebral venous thrombosis; IQR = interquartile range

Number of sinuses involved – Median (IQR)	2 (2 – 3)
1 sinus – N (%)	8/32 (25)
≥2 sinuses – N (%)	24/32 (75)
CVT location	N (%)
Transverse sinus	26/32 (81)
Sigmoid sinus	19/32 (59)
Sagittal superior sinus	14/32 (44)
Sagittal inferior sinus	3/32 (9)
Straight sinus	6/32 (19)
Cavernous sinus	2/32 (6)
Concomitant involvement of ≥1 cortical vein	2/32 (6)
Concomitant involvement of ≥1 deep cerebral vein	6/32 (19)
Venous ischemia – N (%)	10/32 (31)
Hemorrhagic complications – N (%)	6/32 (19)
Parenchymal hemorrhage	5/32 (16)
Subarachnoid hemorrhage	1/32 (3)

Clinicoradiological associations

As the concerned gender, women had a higher number of involved sinuses compared to male patients (median for female 2, IQR 2-3 vs. 1 for male, 1-3; p=0.07). Considering headache characteristics (Table 2), patients with holocranial pain showed a higher prevalence of VI in comparison to those with bilateral (“band-like”) and unilateral headache (50% vs. 20% vs. 0%, respectively; p=0.027). Patients with concomitant neurological defects had more often VI (50% vs 15%; p=0.049) and superior sagittal sinus (SSS) thrombosis (67% vs. 30%, p=0.043). Finally, patients presenting with vomit showed more frequent involvement of the straight sinus (SS, 67% vs. 8% without vomit; p=0.005). All tested associations are reported in Table 4.

**Table 4:** Clinicoradiological associations IH = intracranial hypertension, IQR = interquartile range, PH = parenchymal hemorrhage, SAH = subarachnoid hemorrhage, VI = venous ischemia, + = present, - = absent P = test p of multivariate logistic regression model

		Demographic and clinical characteristics																																								
		Sex			Age			Presentation with headache			Isolated headache			Chronological presentation			Headache intensity				Headache location				Persistent and refractory			Decreased consciousness			≥ 1 sign/symptom of IH			Focal neurological defects			Seizures			Vomit		
		Male (N=8)	Female (N=24)	P	<40 (N=15)	>40 (N=1)	P	+ (N=27)	- (N=5)	P	+ (N=7)	- (N=20)	P	Acute (N=5)	Subacute or chronic (N=22)	P	Mild (N=7)	Moderate (N=11)	Severe (N=9)	P	Unilateral (N=10)	Bilateral (N=5)	Holocranial (N=12)	P	+ (N=8)	- (N=19)	P	+(N=2)	- (N=30)	P	+ (N=7)	- (N=25)	P	+ (N=12)	- (N=20)	P	+ (N=2)	- (N=30)	P	+ (N=6)	- (N=24)	P
Neuroimaging findings	Number of involved sinuses median (IQR)	1.0 (1.0 – 2.8)	2.0 (2.0 – 3.0)	0.07 0	2.0 (2.0 – 3.0)	2.0 (1.0 – 3.0)	0.411	2 (2.0 – 3.0)	1 (1.0 – 2.5)	0.150	2.0 (2.0 – 3.0)	2.0 (2.0 – 3.0)	0.725	3.0 (1.5 – 3.0)	2.0 (2.0 – 3.0)	0.564	2.0 (1.0 – 2.0)	2.0 (2.0 – 3.0)	3.0 (2.0 – 3.0)	0.264	2.5 (2.0 – 3.0)	3.0 (1.5 – 3.0)	2.0 (1.0 – 2.75)	0.182	2.0 (2.0 – 3.0)	2.0 (2.0 – 3.0)	1.000	2.0 (1.0 -3.0)	2.0 (1.75 – 3.0)	0.907	2.0 (2.0 – 3.0)	2.0 (1.0 – 3.0)	0.324	2.5 (1.25 – 3.0)	2.0 (1.25 – 3.0)	0.501	1.50 (1.0 – 1.50)	2.0 (1.75 – 3.0)	0.327	3.0 (1.0 – 3.0)	2.0 (1.75 – 3.0)	0.464
	Concomitant involvement of cortical veins N(%)	1 (12.5)	1 (4.2)	0.444	1 (6.7)	1 (5.9)	1.000	1 (3.7)	1 (20.0)	0.212	0 (0.0)	1 (5.0)	1.000	0 (0.0)	1 (4.5)	1.000	0 (0.0)	1 (9.1)	0 (0.0)	0.470	1 (10.0)	0 (0.0)	0 (0.0)	0.414	1 (12.5)	0 (0.0)	0.296	1 (50.0)	1 (3.3)	0.123	0 (0.0)	2 (8.0)	1.000	1 (8.3)	1 (5.0)	1.000	0 (0.0)	2 (6.7)	1.000	1 (16.7)	1 (3.8)	0.345
	Concomitant involvement of deep cerebral veins N(%)	1 (12.5)	5 (20.8)	1.000	3 (20.0)	3 (17.6)	1.000	6 (22.2)	0 (0.0)	0.555	2 (28.6)	4 (20.0)	0.633	1 (20.0)	5 (22.7)	1.000	1 (14.3)	2 (18.2)	3 (33.3)	0.606	1 (10.0)	3 (60.0)	2 (16.7)	0.074	3 (37.5)	3 (15.8)	0.319	0 (0.0)	6 (20.0)	1.000	1 (14.3)	5 (20.0)	1.000	2 (16.7)	4 (20.0)	1.000	1 (50.0)	5 (16.7)	0.345	3 (50.0)	3 (11.5)	0.063
	Superior sagittal sinus N(%)	2 (25.0)	12 (50.0)	0.412	9 (60.0)	5 (29.4)	0.082	12 (44.4)	2 (40.0)	1.000	1 (14.3)	11 (55.0)	0.091	4 (80.0)	8 (32.4)	0.139	2 (28.6)	5 (45.5)	5 (55.6)	0.557	6 (60.0)	2 (40.0)	4 (33.3)	0.445	4 (50.0)	8 (42.1)	1.000	1 (50.0)	13 (43.3)	1.000	3 (42.9)	11 (44.0)	1.000	8 (66.7)	6 (30.0)	0.043	1 (50.0)	13 (43.3)	1.000	3 (50.0)	11 (42.3)	1.000
	Inferior sagittal sinus N(%)	0 (0.0)	3 (12.5)	0.555	2 (13.3)	1 (5.9)	0.589	3 (11.1)	0 (0.0)	1.000	0 (0.0)	3 (15.0)	0.545	0 (0.0)	3 (13.6)	1.000	0 (0.0)	2 (18.2)	1 (11.1)	0.489	0 (0.0)	2 (40.0)	1 (8.3)	0.062	1 (12.5)	2 (10.5)	1.000	0 (0.0)	3 (10.0)	1.000	1 (14.3)	2 (8.0)	0.536	1 (8.3)	2 (10.0)	1.000	1 (50.0)	2 (6.7)	0.181	1 (16.7)	2 (7.7)	0.476
	Straight sinus N(%)	1 (12.5)	5 (20.8)	1.000	3 (20.0)	3 (17.6)	1.000	6 (22.2)	0 (0.0)	0.555	2 (28.6)	4 (20.0)	0.633	2 (40.0)	4 (18.2)	0.303	1 (14.3)	1 (9.1)	4 (44.4)	0.141	2 (20.0)	2 (40.0)	2 (16.7)	0.561	3 (37.5)	3 (15.8)	0.319	0 (0.0)	6 (20.0)	1.000	1 (14.3)	5 (20.0)	1.000	3 (25.0)	3 (15.0)	0.647	0 (0.0)	6 (20.0)	1.000	4 (66.7)	2 (7.7)	0.005
	Sigmoid sinus N(%)	4 (50.0)	15 (62.5)	0.684	9 (60.0)	10 (58.8)	1.000	18 (66.7)	1 (20.0)	0.132	6 (85.7)	12 (60.0)	0.363	3 (60.0)	15 (68.2)	1.000	4 (57.1)	8 (72.7)	6 (66.7)	0.792	7 (70.0)	3 (60.0)	8 (66.7)	0.928	4 (50.0)	14 (73.7)	0.375	1 (50.0)	18 (60.0)	1.000	5 (71.4)	14 (56.0)	0.671	7 (58.3)	12 (60.0)	1.000	0 (0.0)	10 (63.3)	0.157	3 (50.0)	16 (61.5)	0.666
	Transverse sinus N(%)	5 (62.5)	21 (87.5)	0.148	13 (86.7)	13 (76.5)	0.659	23 (85.2)	3 (60.0)	0.228	6 (85.7)	17 (85.0)	1.000	4 (80.0)	19 (86.4)	1.000	6 (85.7)	9 (81.8)	8 (88.9)	0.906	10 (100.0)	4 (80.0)	9 (75.0)	0.243	7 (77.5)	16 (84.2)	1.000	2 (100.0)	24 (80.0)	1.000	7 (100.0)	19 (76.0)	0.296	10 (83.3)	16 (80.0)	1.000	1 (50.0)	25 (83.3)	0.345	5 (83.3)	21 (80.8)	1.000
	Cavernous sinus N(%)	1 (12.5)	1 (4.3)	0.444	0 (0.0)	2 (11.8)	0.486	0 (0.0)	2 (40.0)	0.051	0 (0.0)	0 (0.0)	-	0 (0.0)	0 (0.0)	-	0 (0.0)	0 (0.0)	0 (0.0)	-	0 (0.0)	0 (0.0)	0 (0.0)	-	0 (0.0)	0 (0.0)	-	0 (0.0)	2 (6.7)	1.000	0 (0.0)	2 (8.0)	1.000	0 (0.0)	2 (10.0)	0.516	0 (0.0)	2 (6.7)	1.000	0 (0.0)	2 (7.7)	1.000
	VI N(%)	3 (37.5)	6 (25.0)	0.654	3 (20.0)	6 (35.3)	0.444	7 (25.9)	2 (40.0)	0.604	0 (0.0)	7 (35.0)	0.137	1 (20.0)	6 (27.3)	1.000	1 (14.3)	3 (27.3)	3 (33.3)	0.683	0 (0.0)	1 (20.0)	6 (50.0)	0.027	4 (50.0)	3 (15.8)	0.145	2 (100.0)	7 (23.3)	0.073	1 (14.3)	8 (32.0)	0.640	6 (50.0)	3 (15.0)	0.049	0 (0.0)	9 (30.0)	1.000	3 (50.0)	6 (23.1)	0.314
	SAH N(%)	0 (0.0)	1 (4.2)	1.000	0 (0.0)	1 (5.9)	1.000	0 (0.0)	1 (20.0)	0.156	0 (0.0)	0 (0.0)	-	0 (0.0)	0 (0.0)	-	0 (0.0)	0 (0.0)	0 (0.0)	-	0 (0.0)	0 (0.0)	0 (0.0)	-	0 (0.0)	0 (0.0)	-	1 (50.0)	0 (0.0)	0.063	0 (0.0)	1 (4.0)	1.000	0 (0.0)	1 (5.0)	1.000	0 (0.0)	1 (3.3)	1.000	0 (0.0)	1 (3.8)	1.000
	PH N(%)	2 (25.0)	2 (8.3)	0.524	0 (0.0)	4 (23.5)	0.167	3 (42.9)	1 (20.0)	1.000	0 (0.0)	3 (15.0)	1.000	1 (20.0)	2 (9.1)	0.429	1 (14.3)	0 (0.0)	2 (22.2)	0.118	0 (0.0)	1 (20.0)	2 (18.2)	0.429	1 (12.5)	2 (10.5)	0.486	1 (50.0)	3 (10.0)	1.000	0 (0.0)	4 (16.0)	1.000	3 (25.0)	1 (5.0)	1.000	0 (0.0)	4 (13.3)	1.000	2 (33.3)	2 (7.7)	0.524

Treatment and outcome

All patients underwent immediate anticoagulation with low-molecular-weight heparin (LMWH, 91%) or sodium heparin (9%). Thereafter, anticoagulation therapy was continued with warfarin (78%) or LWMH (22%) according to current guidelines for CVT treatment [5]. Specifically, 17 out of 32 patients (54%) were treated with anticoagulants for <12 months while 10 patients (32%) underwent anticoagulation for 12-24 months. Only five patients (16%) with permanent risk factors (genetic thrombophilia, malignancies including a case of essential thrombocythemia) underwent long-term (>2 years) anticoagulation with warfarin. The large majority of patients (85%) showed a favorable functional outcome (mRS=0-1) at the discharge (median time 20 days, IQR 13-56). Headache completely resolved after the acute phase in 59% of patients while it improved greatly at discharge in the remaining patients. At follow-up (median time 25 months, IQR 13-47), a good overall functional outcome (median mRS 0, IQR 0-1) was recorded, and no patients died of CVT. On CT/MR venography (median time from the first CT/MR venography = 36 months, IQR 22-53), complete recanalization was observed in 41% of patients and partial recanalization in 53%. No case of CVT recurrence was recorded during the entire follow-up.

Associations with functional outcome

Univariate logistic analysis showed that increasing age and the presence of VI were the only two variables significantly associated with poor (mRS 2-5) outcome (OR = 1.074, 95% CI 1.001-1.152; p=0.047 and OR = 8.400, 95% CI 1.186-59.493; p=0.033, respectively) at discharge. Women showed a lower frequency of poor outcome in comparison to male patients (12.5% vs. 25%), although female sex was not able to predict good prognosis (OR = 0.238, 95% CI 0.037-1.551; p=0.133). Multivariate analysis confirmed that both increasing age (OR = 1.081, 95% CI 1.004-1.165; p=0.038) and VI (OR = 12.089, 95%, CI 1.141-128.104; p=0.039) were independently associated with mRS ≥2.

## Discussion

We analyzed a cohort of consecutive acute CVT patients admitted to the ED of our hospital over a period of 90 months. Our study outlines some relevant clinicoradiological associations, especially with regard to acute CVT-related headache characteristics, which, to date, are still not univocally defined.

Epidemiologically, the higher prevalence of women (>70%) observed in our population, as well as the distribution of risk factors (mostly gender-specific, e.g., oral contraceptives and pregnancy >50%), are in line with observations from larger cohort studies (e.g. International Study on Cerebral Vein and Dural Sinus Thrombosis - ISCVT [3], Cerebral Venous Sinuses Thrombosis Study - VENOST [12]). Our estimated incidence of CVT turned out to be higher (16 cases/year per million) than expected from prior epidemiological studies (5 cases/year per million people) [4,13-14]. However, data from more recent research support our results, indicating an incidence of 13-15.7 million per year [15-16], perhaps because of more complete ascertainment, suggesting that CVT occurrence could be higher than previously believed. Remarkably, unenhanced head CT scan resulted positive for direct (e.g. cord sign, dense triangle sing) or indirect (e.g. venous ischemia, subcortical hemorrhagic infarction, brain swelling) signs consistent with acute CVT in a great proportion of patients (83%), suggesting great value in the emergency diagnosis of CVT [17-19].

As expected, headache was the most prevalent presenting symptom of acute CVT (84%), although with heterogeneous intensity and location (either holocranial, “band-like,” or hemicranial). Patients presenting with headache tended to be younger in comparison to patients reporting different onsets. This finding may be due to cerebral atrophy in the elderly, attenuating effects of IH, as well as to diminished pain reactivity [6]. Notably, the classical thunderclap onset was never reported. Therefore, an isolated headache as a CVT inaugural manifestation reasonably delayed the first-aid access of about a week compared with focal neurological defects at onset. Despite the heterogeneous intensity and location, the recurrence of some features seems to indicate a more frequent pattern for CVT-related headaches. Head pain was more often subacute (82%), new-onset (67%), or with unusual features in comparison to previous episodes of headache (100%), ipsilateral to the involved sinus when hemicranial (90%), with moderate to severe intensity (70%), persistent and refractory to common analgesics (81%), associated with other neurological signs/symptoms (“headache plus” - 74%). However, the wide spectrum of CVT presentations observed in our population highlights that any recent persisting headache should arouse suspicion, particularly in the presence of an underlying prothrombotic condition, as stated by the current international classification for headache disorders - ICHD criteria [20], to avoid possible delay of treatment initiation.

With reference to neuroimaging findings, the distribution of involved sinuses slightly differed from results of the ISCVT cohort [3], which reported a more frequent involvement of the SSS (>60%). This difference might be explained by the high prevalence (75%) of patients with thrombosis of ≥2 sinuses, probably for the higher rate of genetic/acquired systemic prothrombotic risk factors (>70%) in our population vs. localized brain (e.g. vascular anomalies, central nervous system (CNS) tumors, 0%) or maxillofacial precipitants (e.g. infections or trauma, 13%) which are likelier to cause focal CVT. Head CT signs of VI were found in approximately 30% of patients, aligning to the frequency reported by larger studies [3,5]. Interestingly, patients with VI had a higher incidence of holocranial headache (p=0.027). This clinicoradiological association was observed independently from the concomitant presence of ICH or symptoms of IH, supporting the hypothesis that cortical irritation and inflammation due to VI might consistently contribute to the development of headache in CVT [21-23]. SSS thrombosis was more frequently observed in patients with focal neurological defects (p=0.043), likely for the concomitant involvement (50%) of cortical and/or deep cerebral veins, not allowing the development of adequate collateral outflows [24,25], thus leading to transient dysfunction of eloquent parenchymal areas. The more frequent presence of vomit in patients with straight sinus thrombosis (p=0.05) is a novel finding and may be interpreted as an early sign of IH due to the initial development of impaired cerebrospinal fluid circulation when structures neighboring the III ventricle outlet (e.g. thalami) are involved [1-2,6]. SAH was a rare complication of CVT in our population also (only one case, 3%). Our high rate of venous recanalization (partial/complete in 94% of patients) was also in line with observations from prior cohorts receiving anticoagulation [26-27].

Concerning the prognosis, acute CVT was associated with a good overall functional outcome (median mRS 0, IQR 0-1), without cases of death when properly treated. VI and increasing age were associated with poor functional outcomes at discharge (mRS 2-5), as observed by larger studies [3,5]. Remarkably, neither the clinical presentation nor any specific characteristic of headache (e.g. location, intensity, responsiveness to common analgesics) showed an independent association with functional outcome, suggesting the weak prognostic value of pain characteristics at CVT onset.

Our study has several limitations. First, the small number of enrolled subjects (reflecting disease rarity) might have hampered the inferability of our findings and masked otherwise relevant associations. Second, the retrospective design represents a known source of possible information bias. However, clinical data were mainly obtained from neurological visits carried out by expert neurologists during the hospital stay, thus downplaying possible information biases.

## Conclusions

Our study suggests that acute CVT, although accounting for a small part of all cerebrovascular accidents, could be more frequent than previously reported. We confirmed and enriched available data on acute CVT clinicoradiological profiles in a consecutive series of patients admitted to our ED. New-onset, subacute, moderate to severe, and persistent headache represents a major clinical red flag that should prompt urgent investigation for CVT, especially in the presence of concomitant neurological defects and/or well-known risk factors. Unenhanced head CT scan showed great sensitivity as the first-line ED diagnostic investigation, followed by CT/MR venography. Prompt and adequate recognition and management led to an excellent outcome in most of the patients (>85%), whereas older age and presence of VI were associated with poor functional outcomes. Given the rarity of CVT, a disease registry should be implemented to achieve a better and univocal characterization of acute CVT clinicoradiological profile in order to favor early diagnosis and the identification of robust prognostic predictors, which still represent an important challenge.
